# Guideline Adherence in Outpatient Clinics for Chronic Obstructive Pulmonary Disease: Results from a Clinical Audit

**DOI:** 10.1371/journal.pone.0151896

**Published:** 2016-03-17

**Authors:** Jose L. López-Campos, Maria Abad Arranz, Carmen Calero-Acuña, Fernando Romero-Valero, Ruth Ayerbe-García, Antonio Hidalgo-Molina, Ricardo I. Aguilar-Pérez-Grovas, Francisco García-Gil, Francisco Casas-Maldonado, Laura Caballero-Ballesteros, María Sánchez-Palop, Dolores Pérez-Tejero, Alejandro Segado, Jose Calvo-Bonachera, Bárbara Hernández-Sierra, Adolfo Doménech, Macarena Arroyo-Varela, Francisco González-Vargas, Juan J. Cruz-Rueda

**Affiliations:** 1 Unidad Médico-Quirúrgica de Enfermedades Respiratorias, Instituto de Biomedicina de Sevilla (IBiS), Hospital Universitario Virgen del Rocío/Universidad de Sevilla, Seville, Spain; 2 CIBER de Enfermedades Respiratorias (CIBERES), Instituto de Salud Carlos III, Madrid, Spain; 3 Hospital Puerta del Mar, Cádiz, Spain; 4 Hospital Juan Ramón Jiménez, Huelva, Spain; 5 Hospital Universitario Reina Sofía, Córdoba, Spain; 6 Hospital Universitario San Cecilio, Granada, Spain; 7 Hospital Infanta Margarita, Cabra, Córdoba, Spain; 8 Hospital Torrecárdenas, Almería, Spain; 9 Hospital Regional Universitario de Málaga, Málaga, Spain; 10 Hospital Universitario Virgen de las Nieves, Granada, Spain; University of Athens Medical School, SWITZERLAND

## Abstract

**Objectives:**

Previous clinical audits of COPD have provided relevant information about medical intervention in exacerbation admissions. The present study aims to evaluate adherence to current guidelines in COPD through a clinical audit.

**Methods:**

This is a pilot clinical audit performed in hospital outpatient respiratory clinics in Andalusia, Spain (eight provinces with more than 8 million inhabitants), including 9 centers (20% of the public centers in the area) between 2013 and 2014. Cases with an established diagnosis of COPD based on risk factors, clinical symptoms, and a post-bronchodilator FEV_1_/FVC ratio of less than 0.70 were deemed eligible. The performance of the outpatient clinics was benchmarked against three guidance documents available at the time of the audit. The appropriateness of the performance was categorized as excellent (>80%), good (60−80%), adequate (40−59%), inadequate (20−39%), and highly inadequate (<20%).

**Results:**

During the audit, 621 clinical records were audited. Adherence to the different guidelines presented a considerable variability among the different participating hospitals, with an excellent or good adherence for symptom recording, MRC or CAT use, smoking status evaluation, spirometry, or bronchodilation therapy. The most outstanding areas for improvement were the use of the BODE index, the monitoring of treatments, the determination of alpha1-antitrypsin, the performance of exercise testing, and vaccination recommendations.

**Conclusions:**

The present study reflects the situation of clinical care for COPD patients in specialized secondary care outpatient clinics. Adherence to clinical guidelines shows considerable variability in outpatient clinics managing COPD patients, and some aspects of the clinical care can clearly be improved.

## Introduction

Clinical audits have emerged as an overarching tool to measure the adequacy of clinical practice in a given health care setting and temporal context. Far from being simply a collection of information, clinical audits have shown their role in highlighting the gaps between the health care that patients receive and the recommended practices [1]. Accordingly, audits and feedback are being used to improve health care across all areas and to evaluate clinical care for different diseases and in different health care settings [2].

COPD is a disease of the first magnitude in terms of morbidity and mortality [3], with a wide prevalence in the population [4] and a considerable impact on the health system [5, 6]. It follows that health care of the patient with COPD should employ the highest quality standards for its potential impact on the lives of patients. Accordingly, COPD is one of the key diseases in which clinical audits have a special relevance.

Until the last few years, clinical audits for COPD were not very common. The United Kingdom [7], recently followed by Spain [8, 9], has been leading the audit process in COPD over the last several decades. Additionally, several countries have recently started their own audit projects [10–12], and very recently, a European Clinical COPD Audit was carried out in 13 European countries [13, 14]. These audits have provided relevant information about medical intervention in a hospital ward for patients admitted with COPD exacerbation [14], the resources available [15], and the interrelationship between resources and clinical practice [16]. However, there is virtually no experience in auditing COPD patients’ care in an outpatient facility, with only one preliminary attempt recently carried out in Italy [17].

The information regarding how COPD patients are treated in outpatient settings would provide very relevant information on the process of care and might show important areas of improvement that could complete the picture obtained in the hospital setting. Based on our previous audit experiences, we have developed a clinical audit for COPD patients in the outpatient setting [18]. In the present manuscript, we describe the main results of a clinical audit in terms of guideline adherence in outpatient clinics of specialized secondary care centers. The results of this audit will serve to set the scenario for an improvement in health care for COPD patients.

## Methodology

This is a pilot clinical audit performed in hospital outpatient respiratory clinics in the region of Andalusia, Spain (eight provinces with more than 8 million inhabitants). The methodology has been extensively reported previously [18]. Briefly, 20% of centers in the area were invited to participate in this audit. The selection of centers was based on participation in previous audits and was voluntary. Participating centers were categorized as regional hospitals (Highly ranked hospitals offering all medical specialties and providing care to the entire regional population), specialty hospitals (Hospitals offering a higher number of medical specialties than district hospitals and providing care to the entire province in which they are located), and district hospitals (Hospitals offering basic medical specialties and providing care to the population of the town in which they are located, including close villages located at a maximum of 1 hour away). As a pilot study, randomization was not performed, and, therefore, we did not aim to achieve a representative sampling.

Cases with an established diagnosis of COPD based on risk factors, clinical symptoms, and a post-bronchodilator FEV_1_/FVC ratio of less than 0.70 were deemed eligible [19]. Because our goal was to assess the usefulness of formally scheduled regular follow-up visits, only cases with at least 1 year of follow-up were included in the audit. Patients who underwent a first diagnostic visit or presented with an exacerbation were not eligible. Similarly, subjects with significant respiratory comorbidities that could have an impact on the COPD treatment approach were excluded at the local investigator’s discretion.

Based on our previous experience, we estimated that 80 cases per center would be required for this pilot study. The 1-year audit took place between October 2013 and September 2014. Recruitment was performed in 4 three-month periods (October–December 2013, January–March 2014, April–June 2014, and July–September 2014). At the beginning of each period, investigators were instructed to identify consecutive COPD cases at the beginning of each trimester until the desired sample size of 20 per trimester was reached.

The performance of the outpatient clinics was benchmarked against clinical guidelines available at the time of the audit. Throughout the study period, two guidelines—GOLD 2013 [19] and the Spanish National Guideline for COPD (GesEPOC) [20]–were widely and uniformly used in Spain. We therefore carefully reviewed the two guidelines to extract the main statements for the purpose of benchmarking the audited performances. We also considered the 2009 Spain Health-Care Quality Standards in COPD, which were active at the time of the audit [21]. The appropriateness of the outpatient performances in relation to such statements was categorized as excellent (>80%), good (60−80%), adequate (40−59%), inadequate (20−39%), and highly inadequate (<20%).

The audit was approved by the Ethics Committee of the Hospital Universitario Virgen del Rocío (code: 2013PI/201). Clinical records were anonymized in the database by assigning a numerical code through an algorithm. No personal information was registered that could be used directly or indirectly to identify an individual. The relationship between the audit code and the clinical history number was kept locally and was the local investigator’s responsibility. Because of the retrospective nature of the study, the anonymization of data, and the lack of active research interventions, the need for informed consent was waived. The Ethics Committee was aware of this circumstance, clearly explained in the protocol, and approved this procedure.

### Statistical Analysis

All computations were performed using the Statistical Package for Social Sciences, version 20.0 (SPSS; IBM Corporation, Somers, NY, USA). Clinical variables are presented as the mean and standard deviations or absolute and relative frequencies, as appropriate. The variability was expressed by using the inter-hospital range (IHR), which represents the highest and lowest mean value from the participant centers. The significance of this variability was explored by the chi-squared test or ANOVA between the different participant centers. The alpha error was set at 0.05.

## Results

During the audit, 621 clinical records from 9 hospitals were audited. The characteristics of the participant centers are summarized in table 1. The majority were big university hospitals with beds ranging from 220 to 1367.

**Table 1:** Characteristics of the participant centers.

Centre	Type	University hospital	Catchment population	Total number of beds
Centre 1	Specialty hospital	Yes	325,723	699
Centre 2	Specialty hospital	Yes	221,436	770
Centre 3	District hospital	No	200,000	220
Centre 4	Regional hospital	Yes	461,555	1197
Centre 5	Regional hospital	Yes	442,523	1200
Centre 6	Regional hospital	Yes	296,868	600
Centre 7	Regional hospital	Yes	270,000	448
Centre 8	Regional hospital	Yes	370,000	1100
Centre 9	Regional hospital	Yes	554,981	1367

The characteristics of the audited cases are summarized in table 2. Cases were typically male, in the seventh decade of life, with a considerable proportion of current smokers, a homogeneous distribution of comorbidities, and moderate to severe lung function impairment.

**Table 2:** Clinical characteristics of the audited cases (n = 621).

	Average*	Inter-hospital range	P-Value^†^
Age (years)	68.3 (9.8)	64.7–69.7	NS
Male gender (n)	527 (84.9)	51.7–94.8	< 0.001
Current smokers (n)	142 (22.9)	13.8–37.5	< 0.001
Tobacco history (pack-year)	54.7 (30.5)	42.5–66.6	< 0.001
Comorbidities (Charlson)	2.15 (1.5)	1.8–2.4	NS
Psychiatric comorbidities (n)	126 (20.3)	12.3–24.1	NS
Cardiovascular comorbidities (n)	163 (26.2)	18.2–37.5	NS
Previous neoplasms (n)	92 (14.8)	8.6–21.0	NS
Time from diagnosis (years)	5.5 (5.9)	1.06–6.9	0.033
Previous hospitalizations (n)	0.9 (1.6)	0.1–1.3	< 0.001
Body mass index (kg/m^2^)	28.2 (5.3)	26.0–29.6	0.009
FVC (%)	74.6 (20.7)	63.7–98.4	< 0.001
FEV1 (%)	51.9 (19.7)	42.7–59.1	0.001

* Average value expressed as the mean (standard deviation) or absolute (relative) frequency depending on the nature of the variable.

^†^ Calculated for the variability between centers using ANOVA or Chi-square, depending on the nature of the variable.

Adherence to recommendations regarding clinical assessment, phenotypes and treatment monitoring is summarized in tables 3–5. Some items were not recorded because they were not included in the audit as per the scientific committee decision. Fulfillment of the different criteria was variable. Even in items with an excellent adherence to the guidelines, the variability of the results, as measured by the IHR range, was considerable. Although most of the items in Q1 and Q2 from table 2 were independently evaluated in most patients, the fulfillment of all the items occurred in a lower proportion of cases. Using CAT and MRC scores and recording tobacco use were relatively high, the former with good adherence and the latter with excellent adherence, also showing an important variability. Unfortunately, the GesEPOC recommendations on disease phenotypes and the GOLD patient stratification were followed with an inadequate adherence. Additionally, the GesEPOC recommendation to use the BODE or BODEx indexes to evaluate severity had a highly inadequate adherence. Regarding treatment monitoring (Q9 from table 2), guideline adherence varied depending on the item. However, the adherence was highly inadequate when considering all four items together for one patient.

**Table 3:** Recommendations regarding the clinical assessments.

Recommendation	Average value*	Inter-hospital range	P value^†^
Q1. GOLD 2013. COPD assessment must consider the following aspects of the disease:			
• Current level of patient’s symptoms: dyspnea recorded	560 (90.2)	69.1–100	< 0.001
• Severity of the spirometric abnormality	510 (82.1)	52.5–100	< 0.001
• Exacerbation risk	556 (89.5)	57.5–98.7	< 0.001
• Presence of comorbidities	Not recorded		
• All three recorded items	437 (70.4)	30.0–95.0	< 0.001
Q2. GOLD 2013. At each visit, inquire about changes in symptoms since the last visit, including:			
• Cough and sputum	551 (88.7)	62.5–100	< 0.001
• Breathlessness	560 (90.2)	69.1–100	< 0.001
• Fatigue	Not recorded		
• Activity limitation	403 (64.9)	24.7–100	< 0.001
• Sleep disturbances	Not recorded		
• All three recorded items	362 (58.3)	22.2–100	< 0.001
Q3. GOLD 2013. GOLD recommends the use of:			
• The Modified British Medical Research Council (mMRC) questionnaire, or	489 (78.7)	33.3–100	< 0.001
• The COPD Assessment Test (CAT).	108 (17.4)	0–93.8	< 0.001
• Any of the two	489 (78.7)	33.3–100	< 0.001

* Average value expressed as the mean (standard deviation) or absolute (relative) frequency depending on the nature of the variable.

^†^ Calculated for the variability between centers using ANOVA or Chi-square, depending on the nature of the variable. Percentages refer to the whole population (n = 621) unless otherwise indicated. GOLD 2013: Global Initiative for Obstructive Lung Disease 2013 [19]; SEPAR 2009: SEPAR Health-Care Quality Standards 2009 [21]; GesEPOC 2012: Spanish National Guideline for COPD [22].

**Table 4:** Recommendations regarding the clinical phenotypes and multidimensional evaluation.

Recommendation	Average value*	Inter-hospital range	P value^†^
Q4. GesEPOC. The clinical phenotype of COPD should be established in all patients:			
• Cases with GesEPOC phenotype established after the visit	294 (47.3)	8.6–95.0	< 0.001
Q5. The impact of COPD on an individual patient combines the symptomatic assessment with the patient’s spirometric classification and/or risk of exacerbations			
• Cases with GOLD classification established	224 (36.1)	3.4–100	< 0.001
• Cases with either GOLD or GesEPOC classifications established	328 (52.8)	10.3–90.0	< 0.001
• Cases with both GOLD and GesEPOC classifications established	95 (15.3)	1.2–49.4	< 0.001
Q6. GesEPOC. The severity of a patient with COPD is determined by the BODE index.	60 (9.7)	0–32.5	< 0.001
Q7. GesEPOC. Alternatively, the BODEx index can be used for patients with mild-to-moderate COPD.			
• Use of the BODEx index in the cohort	145 (23.3)	0–96.3	< 0.001
• Use of the BODEx index in those with post-bronchodilator FEV1 > 50%	24 (15.9)	0–100	< 0.001

* Average value expressed as the mean (standard deviation) or absolute (relative) frequency depending on the nature of the variable.

^†^ Calculated for the variability between centers using ANOVA or Chi-square, depending on the nature of the variable. Percentages refer to the whole population (n = 621) unless otherwise indicated. GOLD 2013: Global Initiative for Obstructive Lung Disease 2013 [19]; SEPAR 2009: SEPAR Health-Care Quality Standards 2009 [21]; GesEPOC 2012: Spanish National Guideline for COPD [22].

**Table 5:** Recommendations regarding treatment monitoring.

Recommendation	Average value*	Inter-hospital range	P value^†^
Q8. GOLD 2013. At each visit, determine current smoking status and smoke exposure:			
• Smoking status verified	591 (95.2)	72.4–100	< 0.001
• Pack-years calculated	558 (89.9)	68.8–100	< 0.001
• Both items evaluated	533 (85.8)	62.1–100	< 0.001
Q9. GOLD 2013 recommends monitoring of:			
• Dosages of various medications: ICS dose recorded (n = 397)	372 (93.7)	50–100	< 0.001
• Adherence to the regimen	327 (52.7)	3.4–92.6	< 0.001
• Inhaler technique	Not recorded		
• Effectiveness of the current regime at controlling symptoms: dyspnea recorded	560 (90.2)	69.1–100	< 0.001
• Side effects of treatment	145 (23.3)	4.9–66.7	< 0.001
• All four items recorded	93 (15.0)	0–44.4	< 0.001

* Average value expressed as the mean (standard deviation) or absolute (relative) frequency depending on the nature of the variable.

^†^ Calculated for the variability between centers using ANOVA or Chi-square, depending on the nature of the variable. Percentages refer to the whole population (n = 621) unless otherwise indicated. GOLD 2013: Global Initiative for Obstructive Lung Disease 2013 [19]; SEPAR 2009: SEPAR Health-Care Quality Standards 2009 [21]; GesEPOC 2012: Spanish National Guideline for COPD [22].

Adherence to recommendations on the diagnostic tests is summarized in table 6. The performance of a spirometry in the audited visit showed good adherence but with considerable variability. Similarly, the performance of this spirometry after a bronchodilator presented an adequate adherence with very high variability. The indication for a CT scan followed the recommendations in all cases except for 9, in which the indication was not specified in the clinical record. The indications for a CT scan to exclude other associated diseases included suspected neoplasm in 32 (56.1%) cases, lung infiltrates in 8 (14.0%) cases, lung transplant evaluation in 5 (8.8%) cases, hemoptysis in 4 (7.0%) cases, pleural diseases in 4 (7.0%) cases, and other in 4 (7.0%) cases. Determination of serum α1-antitrypsin concentrations was clearly inadequate. Monitoring of physical activity had good adherence. However, the performance of a cardiopulmonary test was clearly highly inadequate. In addition, the six-minute walking test was conducted in 110 cases (17.7%) with IHR of 0–82.5%.

**Table 6:** Recommendations regarding the diagnostic tests.

Recommendation	Average value*	Inter-hospital range	P value^†^
Q10. GOLD 2013. Decline in lung function is best tracked by spirometry performed at least once a year	497 (80.0)	30–100	< 0.001
Q11. GOLD 2013. Spirometry should be performed after the administration of an adequate dose of a short-acting inhaled bronchodilator to minimize variability	352 (56.7)	0–94.9	< 0.001
Q12. GOLD 2013. A chest X-ray is valuable in excluding alternative diagnoses and establishing the presence of significant comorbidities	426 (68.6)	19.2–100	< 0.001
Q13. GOLD 2013. Computed tomography (CT) of the chest is not routinely recommended			
• Cases with CT scan performed	96 (15.5)	2.5–37.5	< 0.001
Q14. GesEPOC. Indications for a chest CT scan are (n = 96):			
• Diagnosis of bronchiectasis	14 (14.6)	0–33.3	0.021
• Exclusion of other associated lung diseases	57 (59.4)	0–100	0.001
• Diagnosis and evaluation of emphysema	16 (16.7)	0–100	0.002
• Indication not available	9 (9.4)	0–46.7	< 0.001
Q15. SEPAR 2009. Serum α1-antitrypsin concentrations should be determined for all COPD patients at least once			
• Serum α1-antitrypsin concentrations evaluated at some time point	190 (30.6)	2.5–62.3	< 0.001
Q16. GOLD 2013. Monitoring of physical activity may be more prognostically relevant prognosis than evaluating exercise capacity	403 (64.9)	24.7–100	< 0.001
Q17. SEPAR 2009. Patients with severe or very severe COPD should undergo the following test at least one time: maximal exercise test (n = 174)	1 (0.6)	0–50	< 0.001

* Average value expressed as the mean (standard deviation) or absolute (relative) frequency depending on the nature of the variable.

^†^ Calculated for the variability between centers using ANOVA or Chi-square, depending on the nature of the variable. Percentages refer to the whole population (n = 621) unless otherwise indicated. GOLD 2013: Global Initiative for Obstructive Lung Disease 2013 [19]; SEPAR 2009: SEPAR Health-Care Quality Standards 2009 [21]; GesEPOC 2012: Spanish National Guideline for COPD [22].

Adherence to recommendations regarding treatment is summarized in tables 7 and 8. Anti-tobacco recommendations for active smokers presented good adherence, but with considerable variability. However, adherence to the recommendation for other non-pharmacological treatment was inadequate for the influenza vaccination and exercise recommendations and lower for the pneumococcal vaccination. The type of pneumococcal vaccination was not recorded as per the scientific committee decision. Other treatments, such as long-term oxygen therapy, were not evaluated because the audit did not record blood gas values to assess the correct indication.

**Table 7:** Recommendations regarding non-pharmacological treatment.

Recommendation	Average value*	Inter-hospital range	P value^†^
Q18. GesEPOC. It is recommended to offer all smokers with COPD advice to quit supported by medical/psychological counseling			
• Current smokers receiving anti-tobacco recommendations (n = 142)	107 (75.4)	20–95.8	< 0.001
Q19. SEPAR 2009. Influenza vaccination should be recommended for all COPD patients	269 (43.3)	2.5–93.8	< 0.001
Q20. SEPAR 2009. Pneumococcal vaccination should be offered to patients with severe COPD and to all COPD patients aged 65 years and older			
• Use of pneumococcal vaccine among those with indication (n = 463)	96 (20.7)	0–43.8	< 0.001
Q21. SEPAR 2009. Regular exercise should be recommended for all COPD patients	276 (44.4)	2.5–88.9	< 0.001

* Average value expressed as the mean (standard deviation) or absolute (relative) frequency depending on the nature of the variable.

^†^ Calculated for the variability between centers using ANOVA or Chi-square, depending on the nature of the variable. Percentages refer to the whole population (n = 621) unless otherwise indicated. GOLD 2013: Global Initiative for Obstructive Lung Disease 2013 [19]; SEPAR 2009: SEPAR Health-Care Quality Standards 2009 [21]; GesEPOC 2012: Spanish National Guideline for COPD [22].

**Table 8:** Recommendations regarding pharmacological treatment.

Recommendation	Average value*	Inter-hospital range	P value^†^
Q22. GesEPOC. Long-acting bronchodilators should be used as first-line treatment in all patients with chronic symptoms	590 (95.0)	86.4–100	0.001
Q23. GesEPOC. Combinations of long-acting bronchodilators should be considered for COPD patients with persistent symptoms despite monotherapy			
• Use of long-acting bronchodilator combinations	453 (72.9)	56.8–84.0	0.006
• Use of long-acting bronchodilator combinations among those with indication (n = 102)	33 (32.4)	0–47.6	NS
Q24. GOLD 2013. Long-term treatment with inhaled corticosteroids is recommended for patients with severe and very severe COPD and frequent exacerbations not adequately controlled by long-acting bronchodilators			
• Use of treatment with inhaled corticosteroids	388 (62.5)	52.1–82.7	< 0.001
Q25. GOLD 2013. Long-term monotherapy with inhaled corticosteroids is not recommended in COPD			
• Use of monotherapy with inhaled corticosteroids	1 (0.2)	0–1.3	NS
Q26. GOLD 2013. The use of antibiotics (other than for treating infectious exacerbations of COPD and other bacterial infections) is not currently indicated			
• Cases not using antibiotics	602 (96.9)	93.8–100	< 0.001
Q27. GOLD 2013. There is some evidence that treatment with mucolytics (such as carbocysteine and N-acetyl-cysteine) may reduce exacerbations in COPD patients not receiving inhaled corticosteroids			
• Use of mucolytics	53 (8.5)	0–19.8	< 0.001
• Use of mucolytics among those not receiving inhaled steroids (n = 174)	11 (6.3)	0–22.2	0.023
Q28. GOLD 2013. The phosphodiesterase-4 inhibitor, roflumilast, may also be used in patients with chronic bronchitis and severe and very severe COPD, and frequent exacerbations not adequately controlled by long-acting bronchodilators			
• Use of phosphodiesterase-4 inhibitors	76 (12.2)	6.2–21.0	NS
• Use of phosphodiesterase-4 inhibitors among those with indication (n = 31)	12 (38.7)	0–100	NS

* Average value expressed as the mean (standard deviation) or absolute (relative) frequency depending on the nature of the variable.

^†^ Calculated for the variability between centers using ANOVA or Chi-square, depending on the nature of the variable. Percentages refer to the whole population (n = 621) unless otherwise indicated. GOLD 2013: Global Initiative for Obstructive Lung Disease 2013 [19]; SEPAR 2009: SEPAR Health-Care Quality Standards 2009 [21]; GesEPOC 2012: Spanish National Guideline for COPD [22].

As expected, long-acting bronchodilators (LABD) are widely used in COPD. However, patients using one LABD who had persistent dyspnea received two LABDs in only 32.4% of cases, suggesting a need for improvement in treatment selection. The number of patients with indications for ICS use (severe and very severe disease with frequent exacerbations) was 42 (6.7%), of which all but two were already receiving ICS. Altogether, 62.5% of all cases used ICS. Phosphodiesterase-4 inhibitors were not frequently used even for those patients with indications.

## Discussion

The present study is the first clinical audit on COPD outpatient clinic performance and provides a comprehensive picture of clinical care for COPD patients in this setting. Although it is a non-randomized pilot study, the information presented here reflects the real-life situation of clinical care in the outpatient setting. Adherence to clinical guidelines varies considerably in outpatient clinics managing COPD patients, with strengths and weaknesses in clinical care.

Clinical audits are conceived as tool to summarize the clinical performance of health care over a specified period of time and are aimed at providing information to health professionals to allow them to assess and adjust their performance [2]. In practical terms, health professionals can receive feedback on their performance based on data derived from their routine practice. While it seems intuitive that health care professionals would be prompted to modify their clinical practice if they received feedback that was inconsistent with that of their peers or accepted guidelines, the importance of this change may not be extremely relevant and is influenced by several factors [9]. Accordingly, audits should be followed not only by feedback but also by an implementation program [23].

Although clinical performance has been exhaustively studied during COPD admissions, the situation in outpatient clinics has only been evaluated in one preliminary report in Italy [17]. That study analyzed clinical histories with a limited number of variables and did not analyzed the variability of clinical care. Other studies have evaluated the rates of adherence to GOLD guidelines for COPD treatment among pulmonologists, but exclusively focusing on pharmacological treatment [24]. In the present study, we have done a comprehensive evaluation of the performance of one typical follow-up clinical visit, and, thus, the degree to which the doctor in charge follows and records the recommended information, highlighting the variability of the performance.

There are two important notes to consider regarding clinical audits. The first is related to the importance of adhering to clinical guidelines. Guidelines are general recommendations on how to provide clinical care for a particular condition. However, it is important to remember that these recommendations are not always evidence-based and that the clinical presentation of diseases is normally variable. It is not surprising to find some type of deviation from the actual recommendations for a given condition. However, the adequate degree of this deviation has not been quantified. The second is that, as a retrospective study, an audit can only evaluate the information that the staff has actually provided in writing in the clinical history. Therefore, evaluations conducted that have not been noted in the history appear as not completed in the audit. For instance, a physician may have checked the inhalation technique but did not write it down in the history. In this regard, it is important to highlight the necessity of noting all of the clinical actions and the consequences they may have for clinical care.

In addition to the information provided, this pilot study has also served as a clinical experience to improve the audit methodology for future audits for COPD in this clinical setting. Accordingly, several limitations should be considered. First, randomization would improve the representativeness of the sample. Second, this audit focused on a cross-sectional evaluation of one particular clinical visit. However, there are some items that should be evaluated with wider temporal limits. For instance, spirometry was not performed in all cases, but it is possible that the spirometry was performed in other recent visits. Third, because this was a pilot study, some of the recorded items recommended by the guidelines were not evaluated in the interest of simplifying the protocol, e.g., sleep disturbances, fatigue or blood gas results. Therefore, future audits should carefully select all of the recommendations in the guidelines. Fourth, the present study was not powered to evaluate the impact of the centers or the physicians’ expertise on the results. Future clinical audits should evaluate the importance of resources and doctors’ expertise on clinical outcomes. Finally, it is important to evaluate the impact of guideline adherence on clinically relevant outcomes. Accordingly, a follow-up of patients is needed to evaluate exacerbation rates and survival.

In 2012, the Spanish COPD guideline (GesEPOC) was released with a novel approach to COPD management [22]. After three years, this seems to be the right time to test the adherence to this guideline. Despite the admittedly important contribution of GesEPOC to patient evaluation [6], it seems that the implementation of this guideline is far from optimal. The two main aspects of the guideline, the categorization of patients according to clinical phenotypes and the severity evaluation according to multidimensional BODE or BODEx indexes, are not extremely common in our area. The BODE index is a good quality-of-life marker within the entire spectrum of COPD severity [25, 26]. However, one obvious limitation is the performance of the six-minute walking test needed for the BODE index, and in our area this is rarely performed; the Italian audit noted previously had a similar finding [17]. However, the BODEx index does not require any extra tests [27] and it is equally infrequently used.

Alpha-1 antitrypsin deficiency in COPD patients is another pending issue [28]. Despite its low prevalence [29], the clinical implications of this genetic form of COPD, the possibility of a specific treatment and the accessible noninvasive diagnostic procedure make it one aspect that should be generalized. Accordingly, indications for active case searches have recently been updated in Spain [30]. However, there is disagreement about the determination of this protein in our area. Previous experiences indicate that is possible to screen for this disease [31]. Notably, some initiatives are being discussed including new screening tools, and there is a debate on the appropriateness of including it in newborn screening [32].

The use of chest radiological techniques in COPD is a current source of debate. It is now accepted that chest radiography is of no value in the diagnosis or follow-up of COPD, except for excluding alternative diagnoses and establishing the presence of significant comorbidities. In our cohort, the most common comorbidity detected in a CT scan was lung neoplasms. Of note, recent studies have investigated the role of CT scans in the early detection of lung cancer [33, 34]. In Spain, as in the rest of Europe, the experience confirms the feasibility and efficacy of lung cancer screening using low-dose CT scans [35]. However, current guidelines are not yet clear enough to recommend annual CT scan screening for COPD patients.

The most followed recommendation on non-pharmacological treatment was anti-tobacco recommendations for active smokers. Despite not being optimal, the adherence to the recommendation to quit smoking was higher than previously reported [17]. However, the vaccination recommendation showed worse adherence, especially with the pneumococcal vaccine. In this regard, it is important to highlight the potential of the vaccination to reduce invasive pneumococcal disease [36].

The pharmaceutical treatment recommendations should be followed with caution. These are results from a clinical audit and not a randomized trial, and they reflect real life prescriptions. Currently, there is an important debate on the use of ICS [37]. In Spain, a recent consensus has provided information on the correct use of inhaled corticosteroids in COPD [38]. However, ICSs are still considered to be overused and the situation in our area is not very different from that in other countries [39]. This opens the debate on ICS withdrawal [40, 41]. Interestingly, by the time this audit was performed, there was no clear recommendation on ICS withdrawal in the guideless that we could benchmark.

In conclusion, the present study reflects the situation of clinical care for COPD patients in specialized secondary care outpatient clinics. Adherence to clinical guidelines varies considerably in outpatient clinics managing COPD patients. Despite this variability, some aspects of clinical care can clearly be improved. With this pilot experiment, a nationwide clinical audit is needed to overcome some of the limitations of the pilot audit, which would include the knowledge gained. It is important that this be followed by feedback and an implementation strategy that allows us to finally improve the clinical care being provided.
